# Micro and nano-scale compartments guide the structural transition of silk protein monomers into silk fibers

**DOI:** 10.1038/s41467-022-35505-w

**Published:** 2022-12-21

**Authors:** D. Eliaz, S. Paul, D. Benyamin, A. Cernescu, S. R. Cohen, I. Rosenhek-Goldian, O. Brookstein, M. E. Miali, A. Solomonov, M. Greenblatt, Y. Levy, U. Raviv, A. Barth, U. Shimanovich

**Affiliations:** 1 Department of Molecular Chemistry and Materials Science, Faculty of Chemistry, Weizmann Institute of Science, 7610001 Rehovot, Israel; 2 Department of Biochemistry and Biophysics, Stockholm University, Svante Arrhenius väg 16C, 10691 Stockholm, Sweden; 3 Institute of Chemistry, The Hebrew University of Jerusalem, Edmond J. Safra Campus, Givat Ram, Jerusalem, 9190401 Israel; 4 Neaspec—Attocube Systems AG, Eglfinger Weg 2, Haar, 85540 Munich Germany; 5 Department of Chemical Research Support, Weizmann Institute of Science, 7610001 Re-hovot, Israel; 6 Department of Chemical and Structural Biology, Weizmann Institute of Science, 7610001 Rehovot, Israel

**Keywords:** Self-assembly, Biomaterials - proteins

## Abstract

Silk is a unique, remarkably strong biomaterial made of simple protein building blocks. To date, no synthetic method has come close to reproducing the properties of natural silk, due to the complexity and insufficient understanding of the mechanism of the silk fiber formation. Here, we use a combination of bulk analytical techniques and nanoscale analytical methods, including nano-infrared spectroscopy coupled with atomic force microscopy, to probe the structural characteristics directly, transitions, and evolution of the associated mechanical properties of silk protein species corresponding to the supramolecular phase states inside the silkworm’s silk gland. We found that the key step in silk-fiber production is the formation of nanoscale compartments that guide the structural transition of proteins from their native fold into crystalline *β*-sheets. Remarkably, this process is reversible. Such reversibility enables the remodeling of the final mechanical characteristics of silk materials. These results open a new route for tailoring silk processing for a wide range of new material formats by controlling the structural transitions and self-assembly of the silk protein’s supramolecular phases.

## Abstract (web summary)

By following morphological changes in supramolecular assemblies of silkworm silk, the authors find that while the initial steps of secretion and storage inside the silk gland follow the micelle theory of silk assembly, a phase rearrangement occurs inside microscale spherical structures that gives rise to the formation of nanoscale spherical assemblies.

## Introduction

Silk is a natural protein-based biopolymer with unique mechanical properties and exquisite biocompatibility and biodegradability^1–3^. Natural silks are produced by many insects and arthropods^4,5^. Notably, spider silk and silkworm silk have been the subject of much interest due to the former’s incredible toughness and the latter’s potential for genetic modification and commercial production^6–10^.

The superior physical and biological properties of silk fibers (produced by spiders or silkworms) are achieved by fine control over the phase transitions and the formation of supramolecular assemblies inside the silk gland. These processes span from the protein synthesis region to the spinning duct^4,11^. Generally, silk protein is stored in a silk gland in liquid form as a highly viscous pulp^12,13^. During the spinning process, the protein is transformed from a soluble, largely disordered random coil structure into solid fibers containing ordered intermolecular hydrogen-bonded, *β*-sheet-rich conformations^14,15^. Interestingly, the generation of the fiber from a soluble silk protein feedstock is based solely on the underlying structural (secondary structure) transformations, while the final fiber structure requires external processing (spinning in the duct)^16–23^. The final composition of the natural silkworm silk fiber comprises a silk fibroin core and a sericin (glycoprotein gum) coating. The coating layer, which is added at the final stages of the spinning process, glues two fibroin fibers to form the final composite material^16^. The structural transformations involve the formation of multiple supramolecular phase states of silk fibroin, from soluble monomers, through microscale spherical assemblies, to liquid crystals and nanofibrils^4,17–21^.

The microscale spherical assemblies (hereafter referred to as micro compartments) play a dual regulatory role: they decrease local viscosity, which enables the storage of a highly unstable and aggregation-prone protein solution, and they regulate protein crystallization under applied shear during the spinning process^22,23^. To date, the structural characteristics and conformational transitions of silk protein inside the compartments remain largely unknown. In particular, we have yet to elucidate the true structural form of the protein inside the supramolecular assemblies when stored in the gland, and the process by which this precursor solution is transformed into a hierarchical polycrystalline structure when spun through the duct.

By following the key morphological changes in the supramolecular assemblies of silkworm silk, we were able to determine that the initial steps of silk secretion and storage inside the silk gland do indeed follow the micelle theory^4,17,24^ of silk assembly. This theory suggests that silk protein spontaneously forms spherical micelle-like structures at high protein concentrations and high viscosities. These micelles are then further spun into micronscale silk fibroin fiber^24^. We observed that a phase rearrangement occurs inside the microscale spherical structures, accompanied by the appearance of nanoscale spherical assemblies. In order to clearly delineate the different types of spherical organization of the silk protein in the different assembly stages, we classify these structures as compartments: the micron-scale spherical structures as microcompartments, and the nanoscale spherical assemblies as nanocompartments. Our structural analysis reveals that protein confined in microcompartments preserves its native secondary structure (initial secondary structure of the protein when stored inside the silk gland), as postulated by the micelle theory^4,17,24^. In contrast, the nanocompartments act as a reversible regulatory step that induces the transformation of natively folded protein into a highly ordered structure, rich in *β*-sheets, in which the formation of nanofibrils is an irreversible part of this structural transformation. These results expand our current knowledge of the morphological nature of silk supramolecular structures and the critical role played by the supramolecular phase states in the structural transformations of fiber-forming proteins in generating fibrillar materials with superior physical properties.

## Results and discussion

### Mechanism underlying silk transformation from soluble protein into solid nanofilaments

**Fig. 1:**
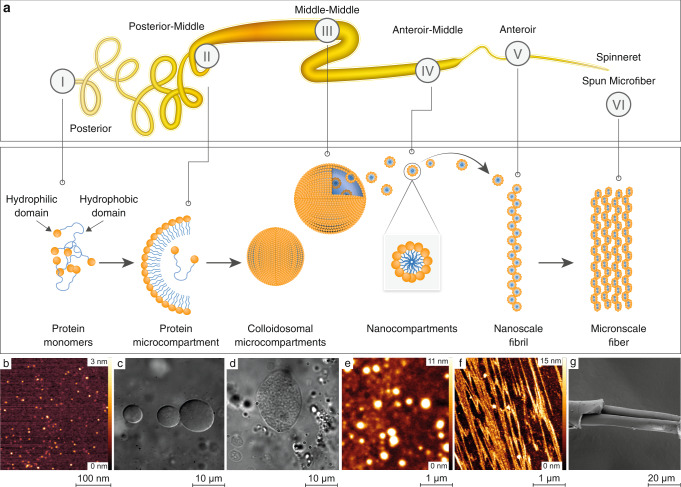
Mechanism underlying silk transformation from soluble protein into solid nanofilaments. **a** Schematic representation of the five regions of the silkworm silk gland (top panel) and their corresponding silk protein supramolecular phases (bottom panel): (i) posterior region, where protein monomers are synthesized, (ii) posterior-middle region, where protein microcompartments were detected, (iii) middle-middle, where appearance of colloidosomal microcompartments was observed, (iv) anterior-middle, where the presence of nanocompartments was detected, (v) anterior region, filled with silk nanofibrils, and (vi) spun microfiber via spinneret. **b** AFM image of protein monomers. **c** Optical microscopy image of microcompartments. **d** Optical microscopy image of colloidosomal microcompartments. **e** AFM image of nanocompartments, **f** AFM image of silk nanofibrils. **g** SEM image of the spun silk microfiber, composed of a fibroin core and a sericin coating layer. The scale bars are shown at the bottom of each image.

To characterize the supramolecular phase states of the fibroin protein inside the silkworm silk gland, we analyzed small (up to 1 cm) sections of silkworm glands from *B. mori* silkworms (see Methods and Fig. 1a) with light microscopy, atomic force microscopy (AFM), and scanning electron microscopy (SEM). The analysis revealed a morphological hierarchy, summarized in Fig. 1. We found that the initial assembly of silk protein, in general, follows the classical micellar theory^4,17,24^, which postulates that when the protein (fibroin) concentration inside the silk gland becomes very high, it triggers the spontaneous formation of the spherical (micelle) micron-scale assemblies that reduce the local viscosity. Namely, protein monomers (Fig. 1b), which are secreted in the posterior part of the gland located close to the silkworm tail (Fig. 1a (i)), first undergo microscale compartmentalization (Fig. 1c) in the posterior-middle part of the silk gland (see silk gland sections in Fig. 1a (ii)). This micron-scale compartmentalization stabilizes the soluble protein pulp and prevents premature aggregation (see AFM, optical images and schematics of the compartments’ composition in Supplementary Figs. 1a, b and 2a). Previous studies, examined the silk fibroin sequence and the spatial alignment of the specific hydrophobic regions (with the repetitive sequence GAGAGAGS) and hydrophilic regions (domains) inside the compartments. They have suggested that larger hydrophilic domains at both the amino and carboxy termini form the outer edges of the microcompartment, sequestering the hydrophobic domains within the compartment’s core^4,25–27^. The smaller hydrophilic domains in between twelve hydrophobic repetitive sequences in the fibroin chain^25–27^ ensure the compartments’ solubility in water, thus preventing premature *β*-sheet formation (see the schematics in Fig. 1 and Supplementary Fig. 2b). As the concentration of fibroin increases, along with small changes in the chemical environment (decrease in pH and changes in ion concentration), the inter-molecular interaction increases, leading to the formation of colloidosomal spherical assemblies (Fig. 1d) and, later on, to their coalescence. These processes take place in the middle-middle (Fig. 1a (iii)) and anterior-middle part of the gland (see Fig. 1a (iv)). The next stage is microcompartment disassembly, which is associated with the conversion of soluble protein into nanofibrillar *β*-sheets rich solid structures (Fig. 1a (v), f). The solid nanofibrils are then aligned with the elongational flow field and spun into microscale fibers (Fig. 1a (vi), g) through the spinneret in the anterior part of the silkworm silk gland^28^.

**Fig. 2:**
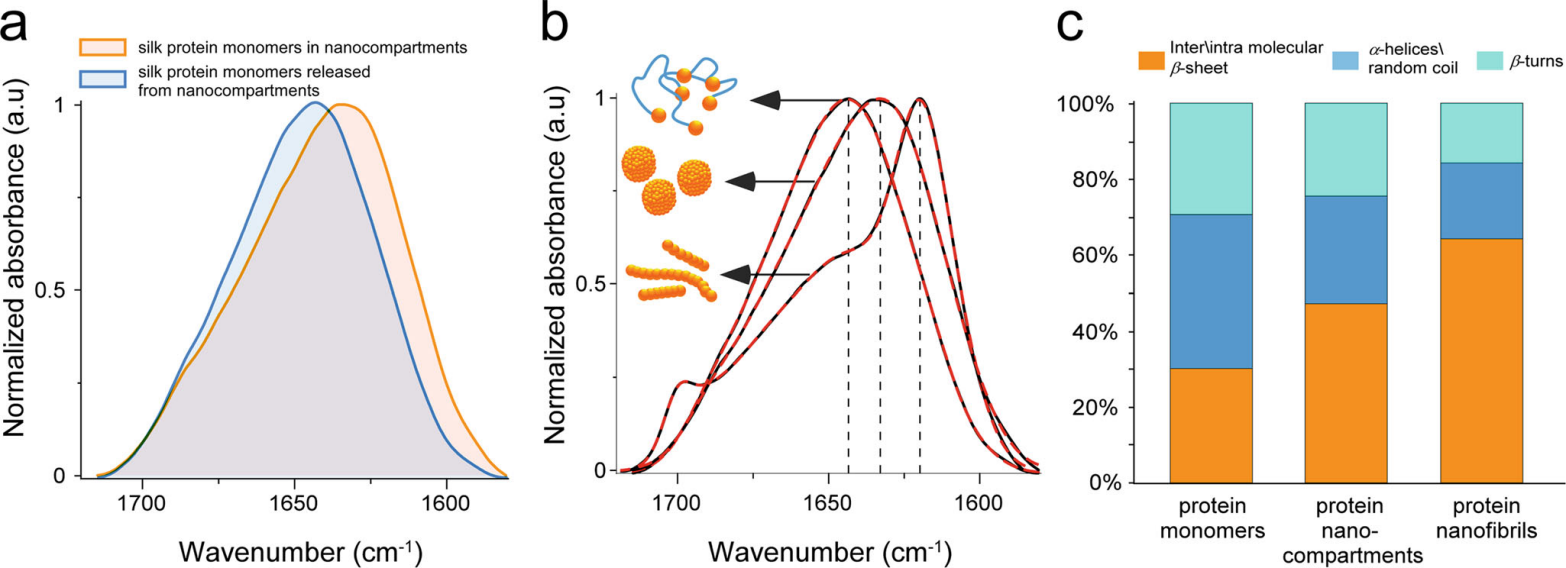
Bulk Fourier Transform Infrared spectroscopy (FTIR) analysis of supramolecular silk fibroin assembly states. **a** Amide I region IR-spectra showing overlap of monomers released from nanocompartments (blue) and monomers within the nanocompartments (orange). The analysis confirms the re-folding event in protein monomers upon nanospherical assembly disintegration. **b** Amide I FTIR spectra of silk fibroin monomers, nanocompartments, and nanofibrils. **c** Comparative analysis of the secondary structure of (**b**) with the band positions of the *β*-sheets at 1610–1635 cm^–1^, anti-parallel *β*-sheets at 1690–1705 cm^–1^, random coil and *α*-helixes at 1635–1665 cm^–1^, and *β*-turns at 1665–1690 cm^–1^. Source data are provided as a Source Data file.

However, our analysis reveals that, in addition to the expected above-described microscale compartmentalization, the proteins undergo a secondary compartmentalization by forming nanoscale spherical structures inside the bigger microscale compartment (Fig. 1a (iii), (iv), Fig. 1d, e; Supplementary Movies 1 and 2). These nanoscale formations are consistent with literature reports^29,30^. The morphological transition takes place in the second half of the middle part of the gland, which is close to the anterior (see Fig. 1a (iv)). Our examination of the stability of these nanoscale compartments (see Methods for details) further revealed a reversibility in the morphological organization. Namely, the nanoscale compartments tend to disassemble and re-assemble. The formation and dissociation of a nanocompartment (with sizes ranging from 20 to 200 nm) is regulated by the protein concentration and/or the amount of water. Thus, an increase in protein concentration above the minimal threshold leads to spontaneous nanocompartment formation (see Supplementary Fig. 3). When the protein concentration decreases, the nanocompartment disassembles into soluble monomeric protein with a native molecular weight of ~400 kDa, composed of a ~390 kDa heavy chain and a ~25 kDa light chain linked by a single disulfide bond^31–33^ (Supplementary Fig. 2c), thus restoring the native protein monomer’s conformation found in the posterior part of the gland, as resolved by Fourier Transform Infrared spectroscopy)FTIR(structural analysis (Fig. 2a).

We next probed the role of protein concentration in spontaneous formation of silk-rich compartments. To this end, we tracked changes in the critical micelle concentration (CMC) using a pyrene-based staining assay^34,35^. Pyrene is a hydrophobic dye that is sensitive to changes in polarity in the environment and is characterized by the presence of several emission peaks in its fluorescence spectra. Changes in the ratio between these peaks occur in response to shifts in the polarity of the surrounding media. We performed a series of silk fibroin dilutions and measured the associated changes in the ratio between I_3_/I_1_ (see Methods). A high ratio of I_3_/I_1_ points to the presence of micellar structures in the solution, thereby confirming the presence of silk compartments. The measured CMC in fibroin nanocompartments was 0.4 mg/ml (Supplementary Fig. 3a). The presence of silk nanocompartments was also confirmed by AFM imaging (Supplementary Fig. 3b). A sigmoid fit confirmed that silk nanocompartments assemble and disassemble as standard micelles at CMC. However, we observed a relatively low slope in the silk nanocompartments^36,37^ (for concentrations between 0.2 mg/ml and 20 mg/ml), indicating a polydispersed compartment size distribution, which is comparable to the behavior of micellar protein *β*-casein^36,37^. Moreover, the low value of silk fibroin CMC suggests that nanocompartments are relatively energetically stable^36,37^. To evaluate the reversibility of spontaneous silk compartmentalization, we diluted highly concentrated compartments containing silk solution (20 mg/ml) down to 0.2 mg/ml and then concentrated them up to 2 mg/ml. The presence and disintegration of the compartments were tracked by measuring changes in I_3_/I_1_ (Supplementary Fig. 3c) and verified by AFM (Supplementary Fig. 3d). The results confirm that the assembly and disassembly of nanocompartments are reversible and concentration-dependent, as has been theorized in previous literature reports^4,24^.

Considering that there is a pH gradient inside the silk gland, as well as shear forces (elongational flow), we further probed the effect of pH and shear on the stability of silk nanocompartments. This was done by exposing fibroin compartments at a concentration of 20 mg/ml to pH values varying between 5.5 and 10 (see Methods; Supplementary Fig. 3e, f). Our observations show that at basic pH values (>pH 8), nanocompartments are stable, while lowering the pH to below pH 7 triggers compartment disintegration, accompanied by structural transitions in the silk protein from the native fold into a highly ordered *β*-sheet-rich conformation. Surprisingly, the exposure of the compartmentalized silk to shear forces (see Methods) did not alter the compartments’ integrity (Supplementary Fig. 3g) or protein fold, an observation consistent with our previous report^12^.

### Structural characteristics of silk phases

Our findings regarding the nature of the silk protein inside the nanocompartments raise three questions: What are the structural characteristics of the protein inside the nanocompartments? What is the role of the formation of nanocompartments in the overall process of protein phase transitions? And what is the impact of the formation of nanoscale assemblies on the mechanics of the silk-based phase states? To address these questions, we performed a conformational study of silk protein assemblies, namely, monomers, nanocompartments, and nanofibrils. This entailed a FTIR analysis, both of bulk assemblies (Fig. 2a, b) and of individual fibrils and monomers by nano-IR imaging and nano-FTIR spectroscopy. In general, the conformational changes in protein structure are characterized by spectral shifts in two major vibrational bands, the amide I (1600–1700 cm^−1^) and the amide II band (1480–1600 cm^−1^), which correspond mainly to C = O stretching vibrations and NH bending/CN stretching vibrations, respectively. The amide I region is commonly used to characterize the intermolecular *β*-sheets (1610–1635 cm^−1^), random coil/ α-helix (1635–1665 cm^−1^), *β*-turn (1665–1690 cm^−1^), and anti-parallel *β*-sheet (1690–1705 cm^−1^) conformations^38,39^.

The comparative FTIR analysis revealed differences between the protein secondary structure of the monomers, microscale and nanoscale compartments and nanofibrils. An increasing *β*-sheet conformation presence was observed in the following order: monomers <nanocompartments <nanofibrils (Fig. 2c). The protein monomers exhibited the lowest fraction of *β*-sheet, and the nanofibrillar assemblies the highest. This is confirmed by the amide I band’s position, which decreases from 1650 cm^−1^ for monomers, via 1630 cm^−1^ for nanomicelles, to 1620 cm^−1^ for nanofibers. This observation indicates that the nanocompartments play a substantial role as structural intermediates in the conformational transition of proteins from a predominantly disordered state to a highly ordered *β*-sheet structure.

Interestingly, our additional analysis revealed that the structural transformations between the supramolecular assembly states are accompanied by changes in the pH and surface charge (ζ-potential)^40^ of the protein complexes. As previously shown^41^, the sequence of silk protein can be roughly classified into three different sections: (1) a repetitive hydrophobic domain with a GAGAGS repetitive motif, which has an isoelectric point of pI = 3.8, (2) a negatively charged hydrophilic N-terminal domain with pI = 4.6, and (3) positively charged domains at the C-terminus with pI = 10.5. Our observations show that the structural transformations of fibroin protein from its monomeric state into nanoscale spherical assemblies as well as into nanofibrils are accompanied by a reduction in the surface charge, driven by a decrease in pH (see Supplementary Fig. 4). We, therefore, performed a series of experiments in which we varied the pH and measured the resultant changes in surface charges (ζ -potential) and in protein folding. The monomers (pH ~9) carry a highly negative surface charge of −20 meV, which creates a repulsive interaction between the residues^42,43^. This, in turn, forces the entire protein to adopt an elongated, molecular conformation. Nanocompartmentalization leads to a reduction in the surface charge (to between −15 and −8 meV), which occurs simultaneously with the change in pH (~6–7), which is cited in the literature^42,43^, and is indicative of the formation of a more compact conformation. Finally, fibrillation, which is caused by lowering the pH, leads to the suppression of dominant repulsive interactions, a phenomenon that promotes a less extended, more compact, *β*-sheet conformation. This observation is in good agreement with the above FTIR analysis, which indicates the presence of a larger fraction of *β*-sheet protein conformations in the silk fibroin nanofibrillar constructs.

### Nano-FTIR analysis of silk assembly states

Even though the conformational FTIR analysis under bulk conditions revealed the general trend of the structural transitions of silk protein assemblies, a major limitation of bulk characterization techniques lies in their inability to differentiate between the protein conformational variability inside nanoscale objects. In particular, as protein self-assembly is a complex, dynamic process, bulk samples of protein fibrils might contain a small fraction of monomers and even nanosized spherical structures. This complicates the interpretation of the results. We, therefore, performed an AFM-based nano-FTIR analysis to determine the local protein conformation in each nm-scale construct: monomers, single nanocompartments and single nanofibrils. Nano-FTIR spectroscopy exploits the enhanced field at the sharp metal tip of the AFM to excite and detect FTIR spectral characteristics simultaneously with nanometer-scale topography. This is a very promising tool for characterizing protein complexes and, as shown recently, fibrillar protein aggregation stages, due to its ability to correlate the information from AFM morphological analyses with FTIR-resolved secondary structures^44,45^. In our analysis and band assignment, we took into account the fact that the bands of the nano-FTIR spectra are upshifted (~10 cm^−1^) compared to the bulk FTIR analysis, due to the technical differences between the two methods of spectra collection (see detailed explanation in the nano-FTIR spectra analysis section in the Supplementary Note 1). Even though we used the standard ranges, we observed the same trend as in the bulk FTIR analysis.

**Fig. 3:**
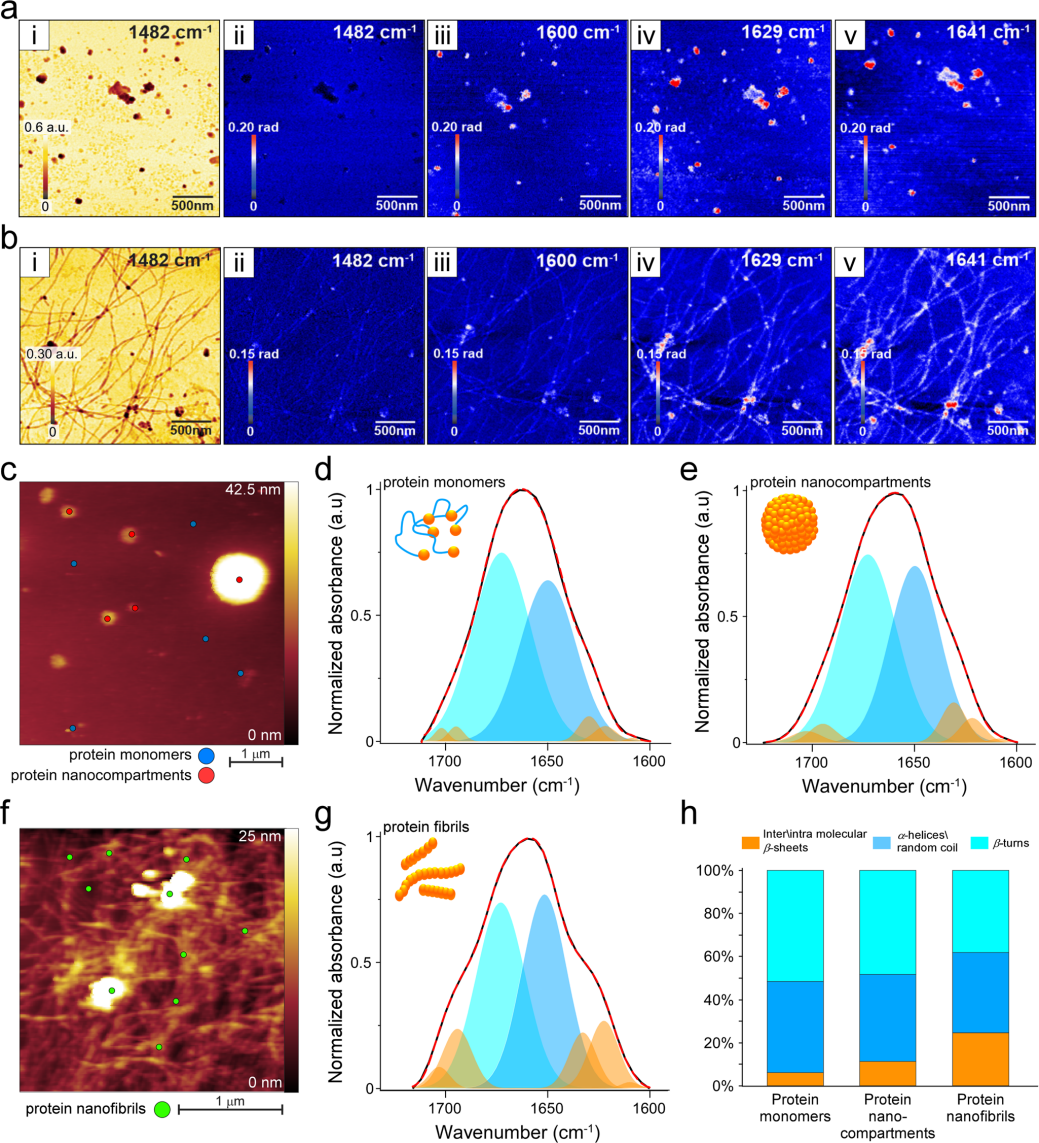
Nano-FTIR analysis of silk protein assemblies. FTIR maps collected from samples containing **a** a mixture of protein nanocompartments and protein monomers and **b** silk protein nanofibrils. (i) FTIR- reflection maps collected at 1482 cm^−1^, to distinguish general protein content from organic contaminants (negative control); (ii) absorption maps collected at 1482 cm^−1^; (iii) absorption maps collected at 1600 cm^−1^; (iv) absorption maps collected at 1629 cm^−1^ showing the β-sheet content; (v) FTIR absorption maps collected at 1641 cm^−1^ showing a signal from a disordered random coil and an α-helix. **c** AFM topography image of silk monomers (marked with blue dots) and silk compartments (marked with red dots), from which IR spectra were recorded and depicted in **d** nano-FTIR spectra of silk protein monomers, and **e** nano-FTIR spectra of protein compartments. **f** AFM topography image depicting silk nanofibrils (marked with green dots), from which FTIR spectra were recorded and depicted in **g**. **h** Bar chart of the relative amounts of the β-sheets at 1610–1635 cm^–1^, the anti-parallel β-sheets at 1690–1705 cm^–1^, random coil and α-helixes at 1635–1665 cm^–1^, and β-turns at 1665–1690 cm^–1^ in (**d**), (**e**) and (**g**). Source data are provided as a Source Data file.

We first performed a qualitative test for nano-FTIR spectra verification on three types of protein assemblies: monomers, nanocompartments, and nanofibrils. As depicted in Fig. 3a (and Supplementary Fig. 5), three wavenumbers have been chosen for this analysis: 1600, 1629, and 1641 cm^−1^. The first is in the “valley” between the amide I and amide II absorption bands, whereas the two other wavenumbers approach the amide I maximum in the nano-FTIR spectra (see below). The phase images are related to the IR absorption and show the expected increase in protein absorption from 1600 to 1641 cm^−1^, associated with a disordered random coil and *α*-helix (Fig. 3a (ii), (iii) and 3b (ii), (iii)). As expected, we observed no signal at 1482 cm^−1^ in any of the samples (see Supplementary Fig. 5), namely, monomers, compartments, and fibrils. The absorption signals at 1600 cm^−1^ confirmed that the structures are proteinaceous. Further analysis elucidated the differences in the fraction of disordered (random coil, 1641 cm^−1^) and ordered (*β*-sheet, 1629 cm^−1^) regions for different protein assemblies (see Fig. 3a, b (iv), (v)).

Next, we conducted a more detailed conformational study using localized nano-FTIR spectra. The nano-FTIR spectra for nanocompartments and monomers were collected from five different locations, and for nanofibrils from ten different locations (Fig. 3c, f, Supplementary Figs. 5 and 6). The spectra were normalized, averaged, and subjected to curve fitting (see Methods). The nano-FTIR analysis revealed a conformational trend akin to bulk FTIR (Fig. 3d, e, g and h). The *β*-sheet content increased from monomers (Fig. 3d) to nanocompartments (Fig. 3e) and from nanocompartments to nanofibrils (Fig. 3g, h).

### The mechanism underlying the silk protein structural transitions

**Fig. 4:**
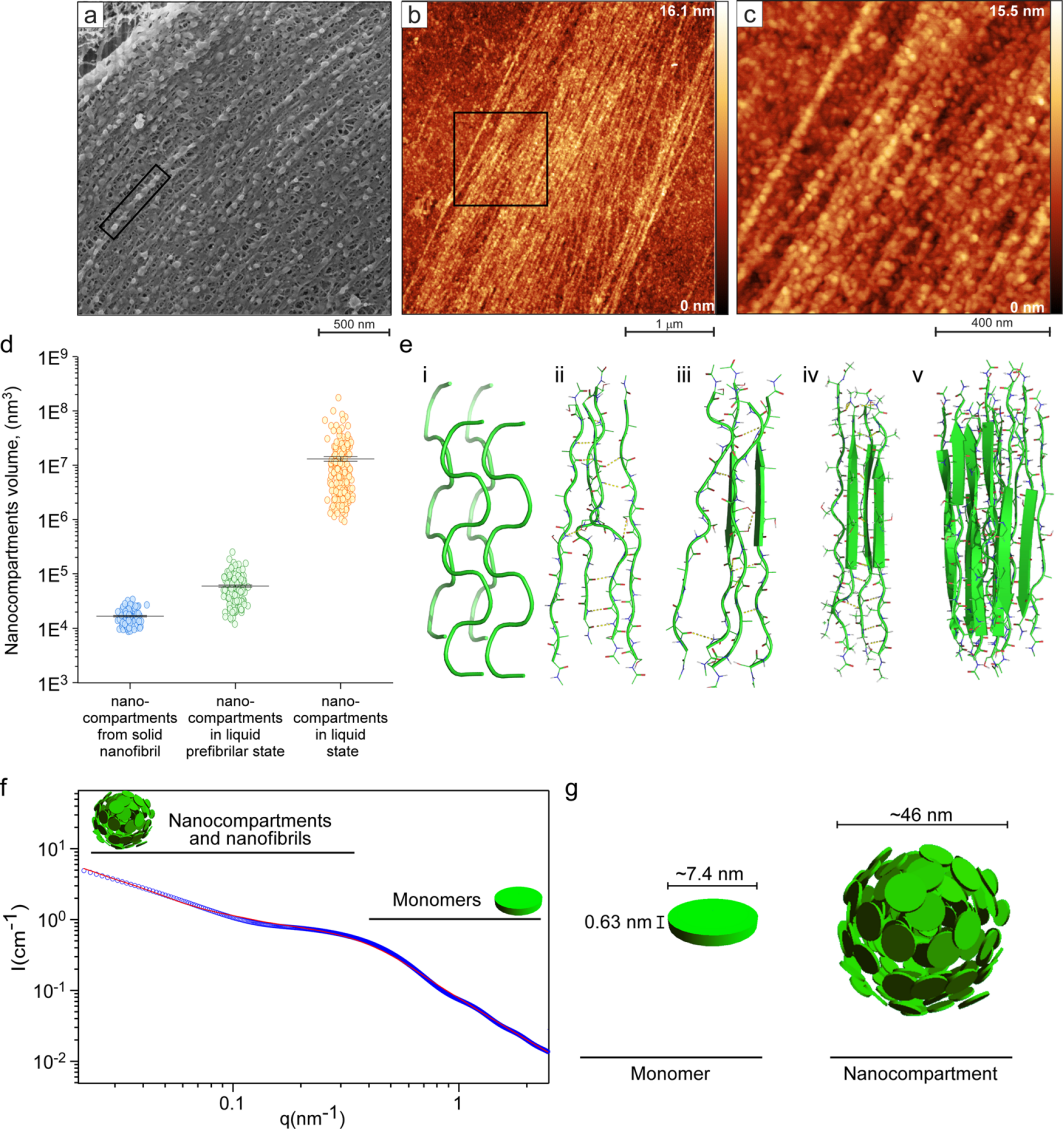
The mechanism underlying the silk protein structural transitions. **a** Freeze-fractured cryo-electron microscopy image of the anterior part of the silkworm silk gland revealing an event of linear ordering of the fibroin nanocompartments (denoted by a black rectangle). **b** AFM image of silk fibroin protein self*-*assembled into nanoscale fibrils. **c** Enlarged image of (**b**) revealing the presence of spherical assemblies in nanofibrillar structures. **d** Volumetric calculation of unordered silk fibroin nanocompartments not subjected to elongational flow stress (average volume of 1.2*10^7^ nm^3^) vs. nanocompartments that have aligned due to shear produced by elongational flow stress (average of 60,160 nm^3^) vs. nanocompartments subjected to shear stress (average volume of 16,750 nm^3^). The standard error and the mean are indicated in the graph. **e** Molecular dynamics simulations of silk fibroin conformational transitions from a relaxed state (representative of an unfolded state) (i) to intermolecular interactions (ii), which gradually induce the formation of *β*-sheets (iii and iv) due to the pulling of the tetrameric silk fibroin hydrophobic domain’s GAGAGS sequence. (v) MD simulation of a 12-meric hydrophobic silk fibroin domain, formed by the pulling of *β*-sheets. **f** SAXS analysis of silk fibroin monomers, nanocompartments and silk fibroin nanofibrils. Azimuthally-interacted background-subtracted solution X-ray scattering absolute intensity, I, as a function of the magnitude of the scattering vector, q, from monomer, nanocompartment and nanofibril- containing samples (open blue symbols). Solid red curves correspond to a linear combination of **g** silk monomers (left illustration) with either elongated (nanofibrils) and spherical (right illustration) structures. Source data are provided as a Source Data file.

To gain further insight into the link between the molecular level mechanisms of protein fibrillation and the supramolecular structural organization, we investigated the changes in the physical characteristics of the protein in the different silk protein phases. The freeze-fracture cryo-scanning electron microscopy (see Methods at Cryo-SEM,) analysis of the anterior part of the silk gland, as well as an AFM analysis of the silk protein extracted from the posterior part of the silk gland^46,47^, revealed that the nanocompartments contracted (Fig. 4a). Nanocompartments in their liquid state (with no exposure to shear forces), whose measured average volume is 1.2*10^7^ nm^3^ and average diameter is 257 nm, shrink under the action of elongational flow stress (Fig. 4a, d and Supplementary Fig. 1a, b). The “prefibrillar” nanocompartments are smaller in volume, featuring an average value of 60,160 nm^3^ and an average diameter of 47 nm (Fig. 4a, d). At the final stage of silk fibrillar assembly, the nanocompartments shrink even further under the action of shear stress and align along the nanofibril axis to form a highly ordered pattern, with repetitive distances of ~30–50 nm (see Supplementary Fig. 7) and an average volume and diameter of 16,750 nm^3^ and 31 nm, respectively (Fig. 4b–d). A volumetric analysis confirmed that nanocompartments tend to shrink in size under applied shear (elongational flow field), accompanied by water expulsion (from ~7% to ~70% of total weight^48–50^) (Fig. 4d).

To gain further insight into the mechanism of silk structural transitions from a relatively disordered state to a more ordered *β*-sheet-rich conformation under the action of the elongational flow field, we performed molecular dynamics (MD) simulations. According to literature reports, the mechanism of silk protein fibrillation is quite complex and is initiated by N-to-N terminus dimerization, followed by hydrophobic interactions between repetitive hydrophobic motifs (GAGAGS)^25–27^. These interactions are believed to be the major driving force that dictates the rate of structural transitions in silk and the kinetics of the protein’s self-assembly. Thus, we performed our computational simulations on the above-mentioned repetitive motifs, which entailed isolating the hydrophobic motif of the silk sequence, known to adopt *β*-sheet folds, and tracking the structural transition. We observed that the conformational transition in silk takes place under tension (Fig. 4e). This is portrayed in Fig. 4e (ii)–(v), which shows how silk protein undergoes various transitions that eventually lead to a long-range *β*-sheet formation from silk hydrophobic motifs.

Seeking to elucidate further the structural composition of silk monomers and silk nanocompartments, we also performed a small-angle X-ray scattering analysis (SAXS) (see Methods). The results are summarized in Fig. 4f, g, and explained in the Methods. The SAXS analysis revealed that silk monomers adopted a disk-like shape (see Fig. 4g) with a radius of 3.7 ± 0.2 nm and polydispersity of 1.2 nm and a height of ~1 nm, which is consistent with our AFM analysis (Supplementary Fig. 1c). Interestingly, we found that for nanocompartment samples, monomers coexisted with spheres (nanocompartments) and fibrillar-like assemblies at a very low mass fraction (see Methods and Supplementary Fig. 8). Whereas the measured dimensions and disk-like shape of the monomer component are identical to the sample containing only silk monomers, the radius of the nanocompartments varied in size.

### Mechanical properties of silk phase states

**Fig. 5:**
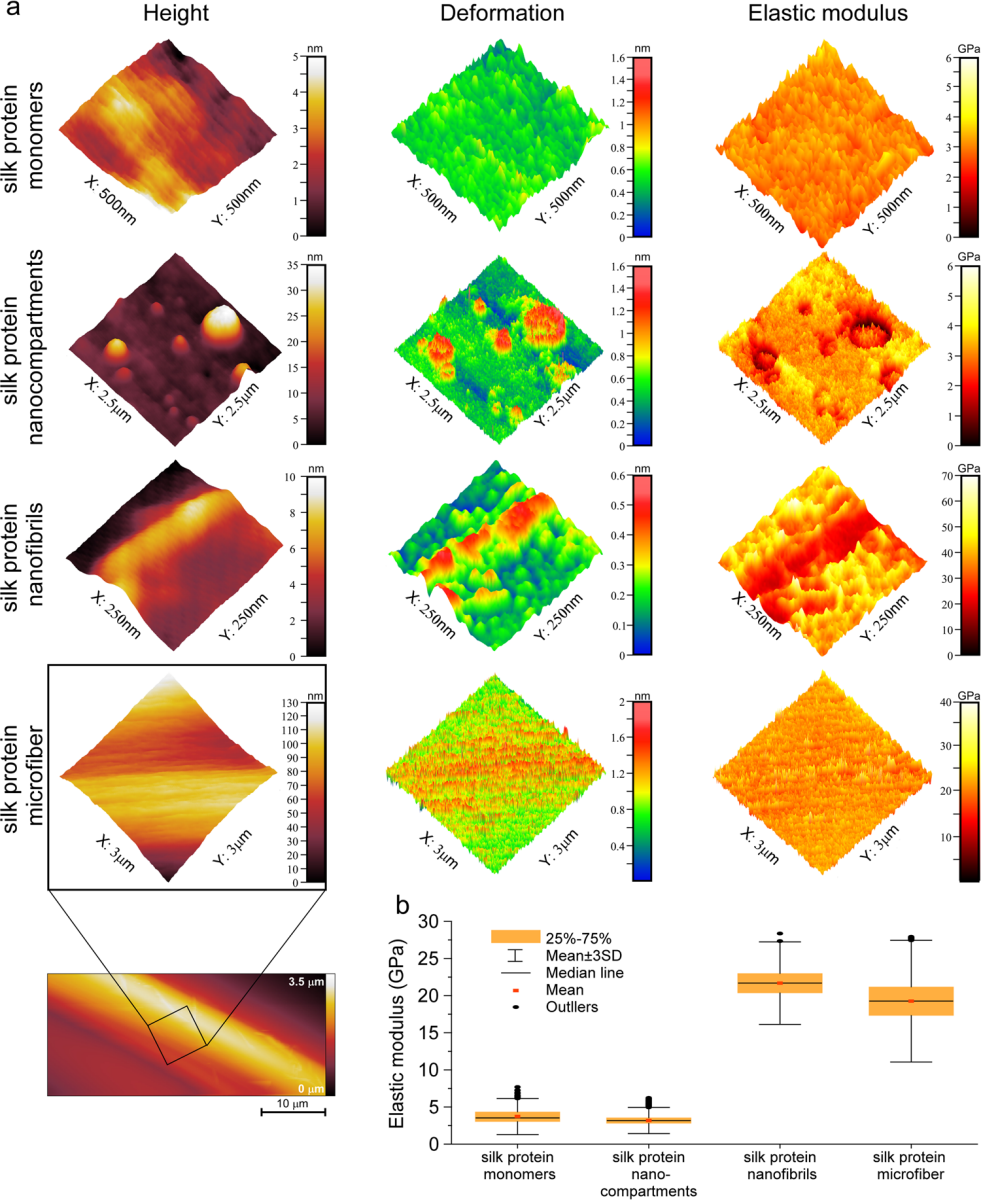
Nanomechanical analysis of silk fibroin assembly states. **a** Comparison of the height, deformation and elastic modulus of silk protein monomers, compartments, nanofibrils and microfibers. **b** Quantitative analysis of the elastic modulus (GPa) of the different assemblies. The elastic modulus rises as the β-sheet content increases. The error bars indicate the standard deviation of the elastic modulus sampled from several AFM images. Source data are provided as a Source Data file.

To better understand the relation between structure and mechanics in silk materials, we studied the nanomechanical properties of the different silk assembly states. The results are shown in Fig. 5 to elucidate the resistance to stress of the monomers, nanocompartments, nanofibrils, and silk microfibers. The Derjaguin-Muller-Toporov (DMT) modulus^51^ was calculated from force curves made at each pixel while keeping the deformation below 10% of the sample thickness, to eliminate the contribution from the mica substrate (see Methods). No significant difference in the elastic modulus was found between the microfiber and nanofibril samples (19.3 ± 2.8 GPa and 21.8 ± 3.2 GPa, respectively). Furthermore, no significant modulus difference was found between monomers and nanocompartments (4.2 ± 0.7 GPa and 3.1 ± 0.7 GPa, respectively). However, there is a pronounced difference in the stiffness of microfibers and nanofibrils compared to the monomers and nanocompartments. Biological samples with a predominant *β*-sheet composition are known for their robust mechanical properties, reflected in their large modulus values^52^. These results, thus, support the findings reported above with respect to the high *β*-sheet content of the microfibers and nanofibrils. In contrast, the markedly lower modulus of monomers and nanocompartments is indicative of protein content with a native fold. Overall, these results suggest that the nanofibers and microfibrils have a similar composition, consisting predominantly of *β*-sheet-conFig.d protein, whereas the monomers and nanocompartments express the mechanically weaker native fold.

In summary, the physical form of proteins within the silkworm silk gland permits their storage at very high concentrations rather than as precipitated material. It also enables their precisely controlled structural transformation from a native form (original conformation) into a *β*-sheet -rich conformation in solution, prior to being formed into solid fibers. We report here important regulatory steps in the silk protein’s transition from a soluble monomeric state, marked by a native, predominantly disordered fold, into insoluble *β*-sheet rich nanofibrils, which results in the formation of nanoscale compartments. Importantly, the nanocompartmentalization step is reversible and enables the un-folding of the protein conformation back to the disordered state. Interestingly, such reversibility can occur before the nanocompartment structural organization is subjected to the shear forces created by elongational flow during the spinning process. Thus, our findings might have implications for the construction of artificial routes for mimicking the exceptional intrinsic mechanical properties of silk fibers.

## Methods

### Silkworm dissection

Domestic monovoltine *Bombyx mori* larvae at their fifth instar were anesthetized with N_2_ for 15 min and then rapidly dissected by removing the head and applying a longitudinal dorsal incision. Silk glands were gently extracted and rinsed with Milli-Q water and then gently placed on a glass slide (25 × 16 mm), which was then set on a microscope stage for microscopy detection. The native silk fibroin (NSF) was extracted from the posterior-midlle and middle-middle parts of the gland.

### Fibroin extraction

Fibroin was extracted from *Bombyx mori* cocoons according to the standard protocol^53^. Briefly, silkworm cocoons were chopped and then boiled in a 20 mM sodium carbonate (≥99.5%, Fischer Chemical, USA) solution at a ratio of 200 ml solution per gram of raw cocoon. The degummed fibers were then washed and dried, followed by their dissolution at 60 °C in a concentrated solution of aqueous lithium bromide. The resultant solution was centrifuged at 12,700 g and dialyzed against Milli-Q water to obtain an aqueous reconstituted silk fibroin (RSF) solution.

### Microscopy analysis

The images and movies were acquired by using Axio observer 7 fluorescent microscope (Carl Zeiss AG, Germany), 20x /0.5 (∞/0.17) microscope objective (Zeiss EC Plan-Neofluar), and Ph2 filter. For the movie acquisition, the exposure time was set to 10 ms, corresponding to 100fps. For the Bright Field (BF) imaging, the lamp was set at 3 kV intensity.

### Atomic force microscopy (AFM)

An aggregated sample drop (50 µl) was placed on freshly cleaved mica and incubated at RT for 5 min. The excess fluid was removed by carefully applying filter paper to the edge of the drop. The mica was washed 3–4 times with water (1 ml in each wash) and dried with a nitrogen stream. Next, the sample was imaged by AFM (JPK Nanowizard 4 AFM (JPK, Germany), with a pixel resolution of 1024 × 1024 (for images with a size of less than 2 x 2 µm) or 2048 × 2048 (for images with a size of 5 x 5 µm).AC240 or AC160 cantilevers (Olympus) were used in AC mode at 23 °C and 35% humidity. Gwyddion 2.61 (64-bit) software was used for the size analysis of the monomers resolved from AFM images: First, the tip’s shape was modeled (based on the manufacturer specifications) and then a surface reconstruction was performed to correct the image by deconvoluting the tip broadening effect on the monomers, after which the radius of 50 monomers was determined using grain analysis.

### Determination of the volume of nanocompartments

ImageJ version 1.53e (64-bit) software was used for the size analysis of the nanocompartments resolved from EM or AFM images. The radius of 50 nanocompartments for each group was determined using grain analysis. The volume was further calculated based on the measured radius.

### AFM nano-mechanical measurements

The samples were prepared by incubation on mica as for the imaging experiments above. Images and mechanical properties were obtained simultaneously using “peak force QNM” on a Multimode AFM (Bruker), under ambient conditions. Tap525A probes, with a nominal spring constant (k) of 200 N/m or RTESP-300-30 (Bruker) with a calibrated spring constant of 47.1 N/m probes were used. The effective tip radius was calibrated on a sample of HOPG with a modulus of 18 GPa or on PolyStyrene (PS) with a modulus of 2.7 GPa. Deformation depths were kept to about 1 nm. The Bruker software (Nanoscope 9.2) was used to compute the elastic modulus maps using the DMT model to fit the force vs. deformation curves.

### Nano-FTIR spectroscopy

The sub-diffraction scattering scanning near-field optical microscope (s-SNOM, Neaspec GmbH. Munich, Germany) uses a metalized AFM tip (Pt/Ir-coated monolithic ARROW-NCPt Si tips from NanoAndMore GmbH, Germany, with a tip radius <10 nm). The tip maps the surface relief (topography) by its basic AFM operation and acts simultaneously as a light-concentrating antenna such that the sample is probed with a nanofocused light field. For nano-IR imaging the tip was illuminated at a specific wavenumber with a quantum cascade laser and for recording nano-FTIR spectra with a broadband laser based on difference frequency generation. The AFM tapping-mode operation (ca. 60 nm amplitude) modulates the near-field interaction between the tip and sample. An asymmetric Michelson interferometer and a lock-in detection of the signal at a higher harmonic of the tapping frequency (~250 kHz) provides a background-free nano-FTIR spectra and nano-FTIR images with spatial resolution imposed by the AFM tip size, independent of the laser wavelength. We used second harmonic to demodulate the signal if not mentioned otherwise. The nano-FTIR spectra were recorded during 5 min with a spectral resolution of 16 cm^−1^. The instrumental response function was removed from the nano-IR spectra by normalizing the measured spectra to a reference Si signal.

### Bulk FTIR spectral measurements

FTIR spectra for the bulk regenerated fibroin were obtained using a Nicolet iS50 FTIR spectrometer equipped with an ATR Smart iTX (attenuated total reflectance) accessory with a resolution of 4 cm^−1^ and 32 individual scans for each measurement. At least three replicate samples were measured for each material studied. The results reported for each sample are comprised of at least three normalized and averaged spectra. For fibril-containing samples, gelled silk was diluted with water and then centrifuged at 16,900 g to obtain a fibril-containing supernatant. Monomer enrichment was performed on freshly extracted silk fibroin 40 mg/ml to 5 mg/ml was used.

### Analysis of IR spectra

All the IR spectra (bulk FTIR and nano-FTIR) between ~1720 and 1600 cm^−1^ were linear baselined to cover the amide I region. To resolve the secondary structures of the protein, the normalized spectra and the replicated spectra from each sample were averaged. Then, the spectra were fitted (by OriginPro 2022 64 bit software) with seven selected Gaussian bands based on refs. 38,39, intermolecular *β*-sheet (1609, 1621, and 1631 cm^–1^), *α*-helix/ random coil (1650 cm^–1^), *β*-turn (1673 cm^–1^), and antiparallel amyloid *β*-sheet (1695 and 1703 cm^–1^) with freedom of ±2 cm^−1^for all the peaks. The fitting analysis of the spectra had to converge, and to reach a Chi-Sqr tolerance value of 1 × 10^−6^. For the bulk FTIR spectral measurements each sample is comprised of at least three normalized and averaged spectra. The measurements obtained from the nano-FTIR spectroscopy analysis were repeated five times for monomers and nanocompartments samples and ten times for fibrils samples. The obtained spectra have been averaged and normalized.

### Silk gland sample preparation for electron microscopy

The silkworm’s anterior gland was cut into pieces using a razor blade and placed between two aluminum discs (Wohlwend Engineering Office, Switzerland) with a depth of 100 µm each. Mili-Q water was used to fill the empty spaces between the sample and the disc walls. The sandwiched sample was frozen in an EM ICE high-pressure freezing (HPF) machine (Leica Microsystems, Vienna, Austria). Samples were kept in liquid nitrogen until processed further.

Frozen samples were mounted on a holder and transferred to a BAF 60 freeze fracture device (Leica Microsystems, Vienna, Austria) using a VCT 100 Vacuum Cryo Transfer device (Leica). After fracturing at a temperature of −120 °C, samples were transferred to an Ultra 55 SEM (Zeiss, Germany) cryo-SEM.

### High-resolution scanning electron microscopy (HRSEM) analysis

HRSEM images of the silk gland were obtained using Ultra-55 using a secondary electron in-lens detector at an acceleration voltage of 1 keV and temperature of −120 °C. Some structures were visualized after sublimation (etching) at −80 °C for 10–20 min. The samples were fixed onto aluminum stubs with carbon tape. SIGMA ultra-high-resolution SEM (Carl Zeiss, Germany) was used to observe silk fiber)without coating) using a backscattered electron detector with at an acceleration voltage of 1.2 keV and with an aperture size of 30 µm. For both microscopes, the chamber vacuum and the gun vacuum conditions were 5*10^−5^ mbar and 1–3*10^−9^ mbar, respectively.

### ζ- potential

The surface charge of the materials was measured by Zetasizer Nano ZSP (Malvern Instruments, UK). Disposable folded capillary cells (catalog number DTS1070) were used for measuring the ζ-potential. The experiments were performed at room temperature (25 °C) with an equilibration time of 25 s. The pH was varied by titration with HCl or NaOH. Each sample was tested three times with 100 runs per single measurement.

### Gel electrophoresis (SDS-PAGE)

25 µg of the silk fibroin sample were loaded and run on a gradient gel (4–20%) from Geba using the manufacture protocol. The gel was stained by InstantBlue® Coomassie Protein Stain (ISB1L) (ab119211) overnight and washed for several hours with water.

### Molecular dynamics simulations

Coordinates and unit cell parameters for the repeating peptide (Ala-Gly)_2_-Ser-Gly were obtained from Okuyama et. al. (2001)^54^. A system containing 2 parallel and 2 anti-parallel strands with 12 residues in each strand was constructed based on the crystal symmetry operators using PyMOL. To avoid charge effects, the N-termi of the chains were acetylated and the C-termi blocked by methyl-amine. The GROMACS program (version 2020.3) was used with the Amber99sb-ILDN force field. The tetramer was placed in an orthorhombic box filled with water molecules and NaCl at 0.125 M. After minimizing the energy, the system was subjected to 100 ps NVT and NPT equilibration regimes. This was followed by 1 ns of pulling dynamics at a constant force of 500 kJmol^−1^nm^−1^. The force direction was parallel to the length of the strands and applied to the center of mass of the 4 terminal atoms on each end of the tetramer (two N-methyl carbon atoms and two acetyl methyl carbon atoms). A similar procedure was used to create a 4 × 3 system of peptides (12 strands: 2 layers of 4 parallel strands that sandwiched a layer of 4 anti-parallel strands). In this case, the pulling force was increased to 3000 kJmol^−1^nm^−1^, and run for 1 nanosec.

### Small-angle X-ray scattering (SAXS) measurements

Solution SAXS measurements were performed at the ID02 beamline at the European Synchrotron Radiation Facility (ESRF), using a beam size of 32.4 × 145 μm^2^ (vertical and horizontal, respectively), photon energy of 12.23 keV, Eiger2 4 M (Dectris AG) detector, sample-to-detector distance of 3.114 m, and exposure time of 0.1 s^55^. SAXS models were computed by X + software, using a water electron density of 333 *e⋅mm*^−3 ^^56^. We fitted the data to a linear combination of uniform disks, and either sphere and/or rod geometries.

We used X + ^57^ for the geometric model of a disk of radius 3.7 ± 0.2 nm, polydispersity of 1.2 nm, and a height of 0.63 ± 0.05 nm. We also used X + for the geometric model of a sphere with a radius 23 ± 2 nm and polydispersity of 1.4 nm. D + software^58^ was used to compute the geometric slab model with a height of 1 nm, a width of 90 ± 20 nm, and a length of 250 ± 50 nm, representing aggregating fibrils. Based on the contribution of the intensities of each model at zero scattering vector and the volume of each model, we computed the mass fraction of disks (0.9871), spheres (0.0003), and fibrils (0.0126) in the modeled red curve, presented in Fig. 4f.

### Determining the changes in CMC of silk nanocompartments in response to changes in fibroin concentration, pH, and shear stress

To determine the CMC, fibroin (RSF) at a concentration of 50 mg/ml and at pH 5.5 was diluted with doubly distilled water (DDW) to form a series of concentrations ranging from 50 mg/ml to 2.4 µg/ml. A stock solution of 5.5 × 10^−5^ M pyrene in 20% ethanol was added to each silk sample in a 1:100 ratio, to reach a final concentration of 5.5 × 10^−7^ M (0.02% ethanol). The fluorescence emission intensity was recorded using a 96-well plate on a micro-plate reader (CLARIOstar of BMG 16 labtech) at room temperature. The excitation wavelength was 336 nm, and the measured emission wavelengths were I_1_ = 371 nm and I_3_ = 381 nm (the first and third peaks of the pyrene emission spectra, respectively). The experiment was repeated three times, and the ratio of I_3_/I_1_ was plotted against the fibroin concentration. The standard deviations are indicated by the error bars on the corresponding Supplementary Fig. 3a^34,59–61^.

To determine the effect of pH on nanocompartments, a stock solution of fibroin (RSF) at a concentration of 20 mg/ml and pH 5.5 was adjusted to pH 6, 7, 8, 9 and 10 using 0.1 M NaOH (minimal concentration change).

To determine the reversibility of the compartmentalization process, namely, the transition of the silk assembly from monomers to nanocompartments and then back to monomers, fibroin at a concentration of 20 mg/ml was diluted to 2 mg/ml and 0.2 mg/ml. Then, diluted samples at the concentration of 0.2 mg/ml were re-concentrated to 2 mg/ml with a Vivaspin 20 Ultrafiltration Unit (with 100,000 MWCO PES of Sartorius, catalog number VS2041). The samples were tested three times using pyrene, as described above.

To determine the effect of shear force on the nanocompartments, fibroin compartments at a concentration of 20 mg/ml and 2 mg/ml at pH 5.5 were vortexed at 500 RPM for 1 h, using Mixing Block MB-102 of Bioer Thermocell. Then, the stability of the nanocompartments was probed immediately by using a pyrene assay three times^62,63^.

### Statistics and reproducibility

The numerical data are expressed as the mean ± s.d. (μ ± σ, *n* ≥ 3 of independent experiments). The experiments throughout this work were repeated at least three times independently with similar results in AFM, Nano-FTIR analysis, Bulk FTIR, SDS-PAGE, HRSEM, ζ-potential and all the pyrene experiments. The analysis presented in Fig. 3c–h is based on five points measured for monomers and for nanocompartments and ten points measured for fibrils. The analysis presented in Fig. 5b is based on measured points of 10,180 monomers, 64,452 nanocompartments, 522 fibrils and 111,816 microfibers. Data are presented as mean values ± s.d (μ ± 3σ). The results presented in Supplementary Fig. 3c, g are from independent experiments, and the dots correspond to the calculated ratio of I_3_/I_1_. For all the experiments, the statistical analysis has been performed by using OriginPro 2022 software.

### Reporting summary

Further information on research design is available in the Nature Portfolio Reporting Summary linked to this article.

## Supplementary information

Supplemetary Information

Description of Additional Supplementary Files

Supplementary Movie 1

Supplementary Movie 2

Reporting Summary

## Data Availability

The datasets generated and analyzed in the current study are available from the corresponding author upon request. Source data are provided with this paper.

## Source data

Source Data
