# Supplementary material for: Micro and nano-scale compartments guide the structural transition of silk protein monomers into silk fibers
Source: Nat Commun. 2022 Dec 21;13:7856. doi: 10.1038/s41467-022-35505-w (PMC9772184; doi:10.1038/s41467-022-35505-w)
Supplement: Supplementary file 1 — Supplemetary Information [file 41467_2022_35505_MOESM1_ESM.pdf]

Micro and nano-scale compartments guide the structural transition of silk protein monomers into silk fibers

D. Eliaz[1], S. Paul[2], D. Benyamin[3], A. Cernescu[4], S. R. Cohen[5], I. Rosenhek-Goldian[5], O. Brookstein[1], M. E. Miali[1], A. Solomonov[1], M. Greenblatt[6], Y. Levy[6], U. Raviv[3], A. Barth[2], and U. Shimanovich[1]*

[1]Department of Molecular Chemistry and Material Sciences, Faculty of Chemistry, Weizmann Institute of Science, 7610001 Rehovot, Israel

[2]Department of Biochemistry and Biophysics, Stockholm University, Svante Arrhenius väg 16C, 10691 Stockholm, Sweden.

[3]Institute of Chemistry, The Hebrew University of Jerusalem, Edmond J. Safra Campus, Givat Ram, Jerusalem 9190401, Israel

[4] Neaspec – Attocube Systems AG, Eglfinger Weg 2, Haar, 85540, Munich, Germany

[5]Department of Chemical Research Support, Weizmann Institute of Science, 7610001 Rehovot, Israel

[6]Department of Chemical and Structural Biology, Weizmann Institute of Science, 7610001 Rehovot, Israel

* To whom correspondence should be addressed: ulyana.shimanovich@weizmann.ac.il.

Keywords: silk fibers, nanospectroscopy, protein self-assembly, β-sheet conformation

Supplementary Information

Includes:

Supplementary Figures

Supplementary Figure 1 - **Morphological and size analysis of silk fibroin monomers and compartments.**

Supplementary Figure 2 - **Spatial alignment od silk fibroin chains in compartments.**

Supplementary Figure 3 - **Mechanism of silk compartments formation.**

Supplementary Figure 4 - **Correlation between the surface charge and pH in silk compartments.**

Supplementary Figure 5 - **Nano-FTIR analysis of silk protein monomers and nanocompartments**.

Supplementary Figure 6 - ***Nano-IR analysis of silk protein assemblies.***

Supplementary Figure 7 - **Silk nanocopartments comprising silk nanodibril.**

Supplementary Figure 8 - **Morphology of protein monomers, nanocompartments and nanofibrils.**

Supplementary Notes

Supplementary Note 1- **Nano-FTIR spectroscopy for secondary structure analysis of proteins**

Supplementary Tables:

Supplementary Table 1

Supplementary Table 2

Supplementary Figures

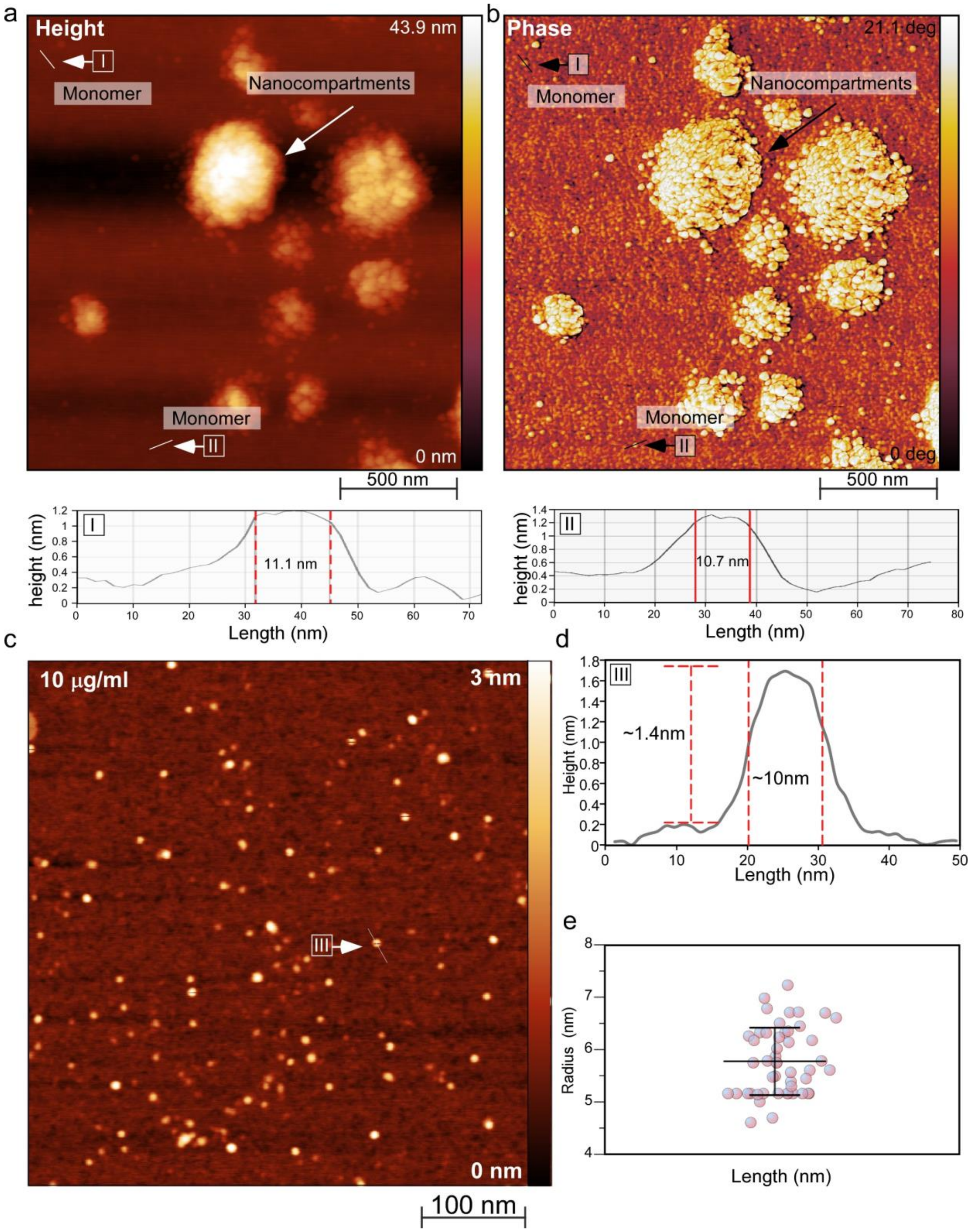

Supplementary Figure 1. Morphological and size analysis of silk fibroin monomers and compartments. *(a)* AFM topography of nanocompartments and silk monomers. Arrows I and II indicate the cross-sectional analysis (see bottom inserts) of monomers. *(b)* Corresponding phase image of the topography image in (*a*). Scale bars are 500 nm. *(c)* AFM image of monomers (scale bar 100 nm) and *(d) a* cross-sectional analysis of a single monomer. *(e)* Analysis of monomers' radii data are presented as mean values ±*s.d* *(μ ± σ, n=50)*. Source data are provided as a Source Data file.

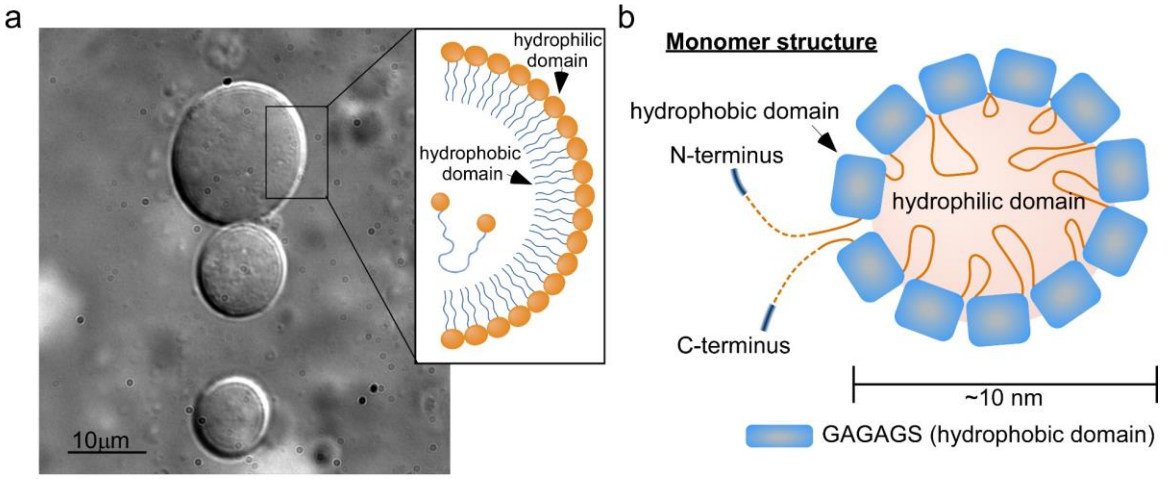

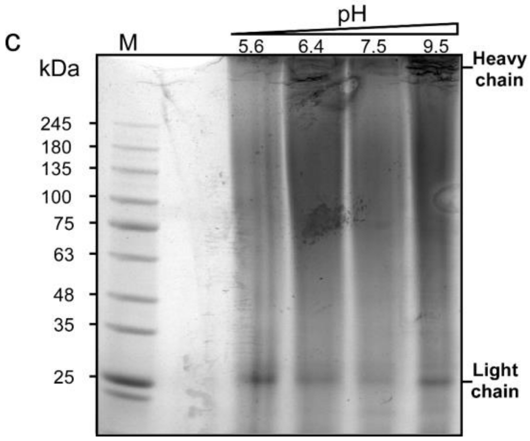

Supplementary Figure 2. Spatial alignment od silk fibroin chains in compartments. *(a)* Optical microscopy image of silk fibroin miconscale compartments, extracted directly from the silkworm gland. The schematic on the right-hand side depicts the alignment of the protein chain inside these compartments, where hydrophilic domains are localized at the interface, sequestering the twelve hydrophobic domains. *(b)* The silk fibroin sequence and the spatial alignment of the specific hydrophobic regions (with the repetitive sequence GAGAGAGS) and the hydrophilic regions (domain). *(c)* SDS gel analysis of the light and heavy chain of silk fibroin at pH 5.6, 6.4, 7.5 and 9.5. Source data are provided as a Source Data file.

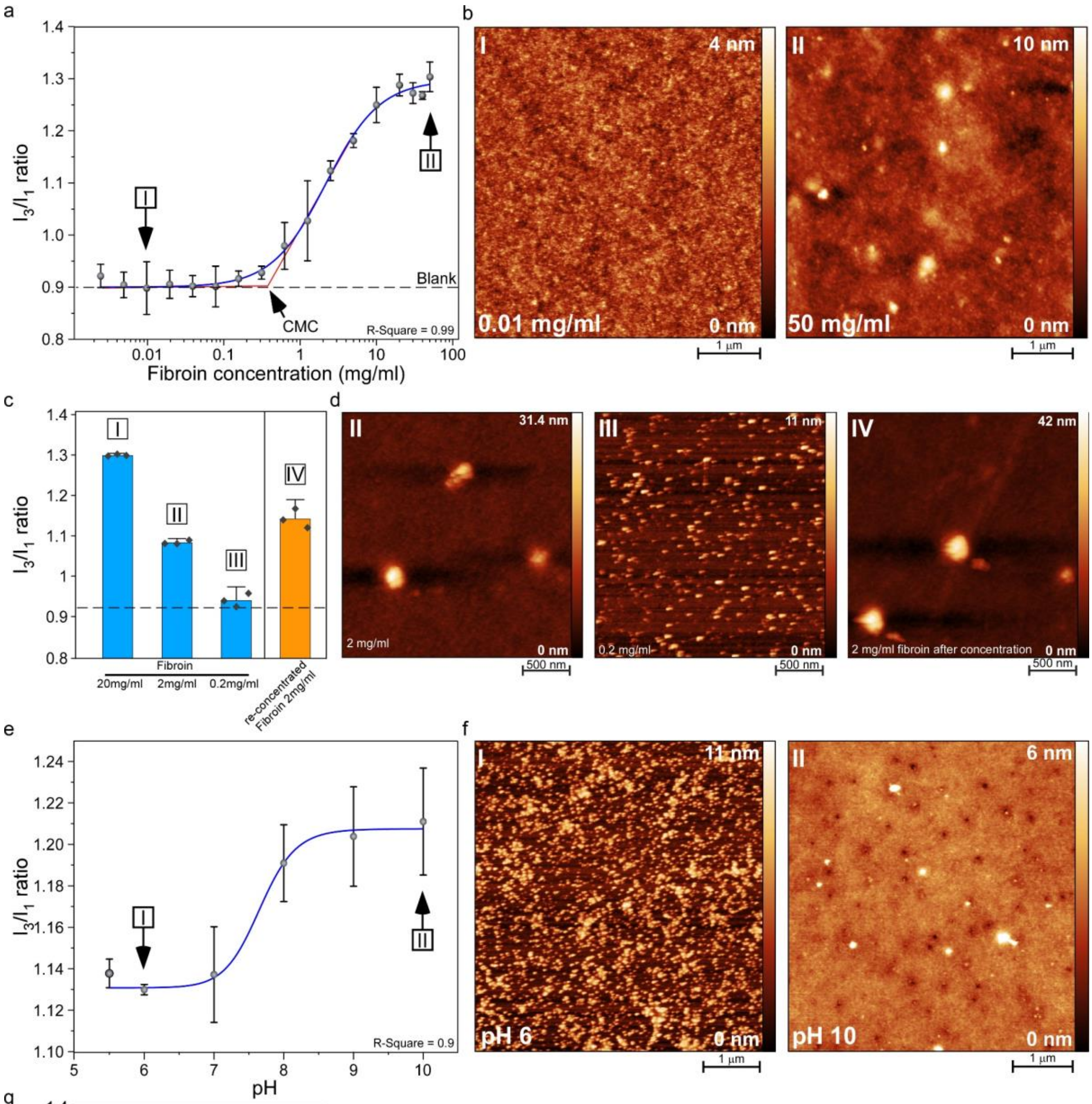

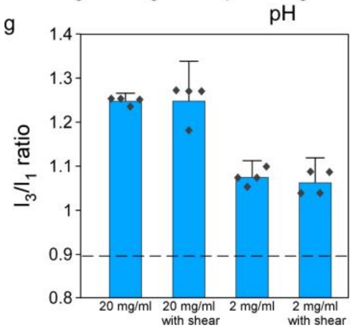

Supplementary Figure 3. Mechanism of silk compartments formation. *(a)* Critical micelle concentration (CMC) assay for fibroin with concentration varying between 50 mg/ml to 2.4 µg/ml. The CMC is determined to be at fibroin concentration of 0.4 mg/ml. Data are presented as mean values ±s.d (µ ± σ, n=3 of independent experiments). The dashed line corresponds to the blank sample (DDW without protein). The two arrows designated as I and II correspond to the silk fibroin samples that were further analyzed by AFM. Source data are provided as a Source Data file. *(b)* The AFM topography images correspond to (I) low fibroin concentration (10 µg/ml) and (II) high fibroin concentration (50 mg/ml). The scale bars for AFM images are 1 µm. *(c)* Compartmentalization reversibility test from monomers to nanocompartments. Three concentrations of fibroin 20 mg/ml (I), 2 mg/ml (II) and 0.2 mg/ml (III) are presented in a blue column and a re-concentrated fibroin (IV) from 0.2 to 2 mg/ml is presented in the orange column. The dashed line indicates the ratio for the blank sample (solution without protein) and the data are presented as mean values ±s.d (µ ± σ, n=3). Source data are provided as a Source Data file. AFM images of 2 mg/ml (II), 0.2 mg/ml (III) and the re-concentrated fibroin (IV) are shown in *(d)*. The scale bars for AFM images are 500 nm. *(e)* The effect of pH ranging between 5.5-10 on the stability of nanocompartments. The data are presented as mean values ±s.d (µ ± σ, n=3 of independent experiments). Source data are provided as a Source Data file. The two arrows correspond to the AFM images of the fibroin solution at pH 6 and at pH 10 that are shown in *(f)*. The scale bars for AFM images are 1 µm. *(g)* The effects of shear force on the stability of nanocompartments. The data are presented as mean values ±s.d (µ ± σ, n=4). Source data are provided as a Source Data file.

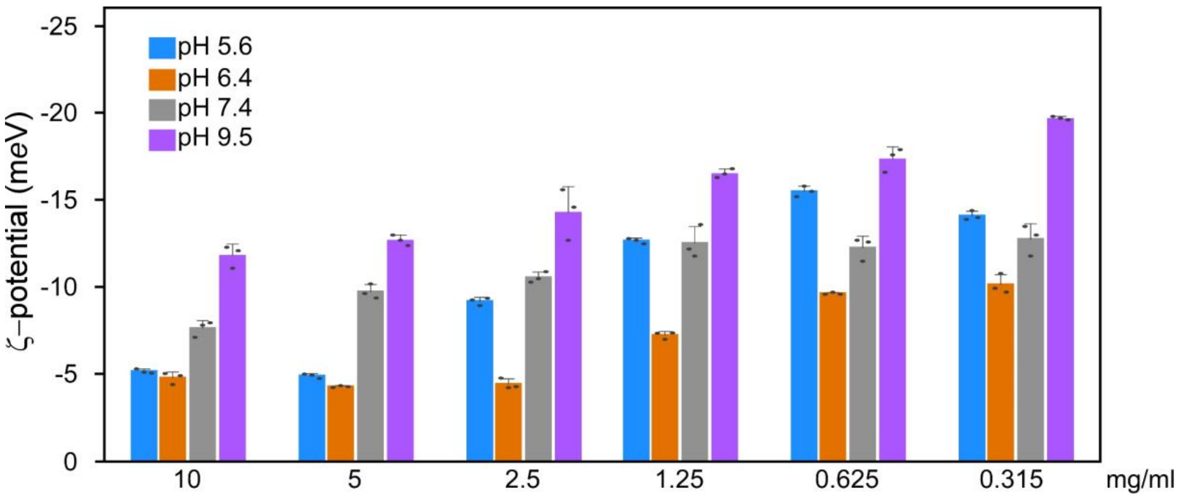

Supplementary Figure 4. Correlation between the surface charge and pH in silk compartments. ζ-potential analysis of silk fibroin at pH 5.6, 6.4, 7.4 and 9.5 at different concentrations: 10, 5, 2.5, 1.25, 0.625, and 0.315 mg/ml. The data are shown as mean values ±s.d (μ ± σ, n=3 of independent experiments and the dots correspond to the actual measured data). Source data are provided as a Source Data file.

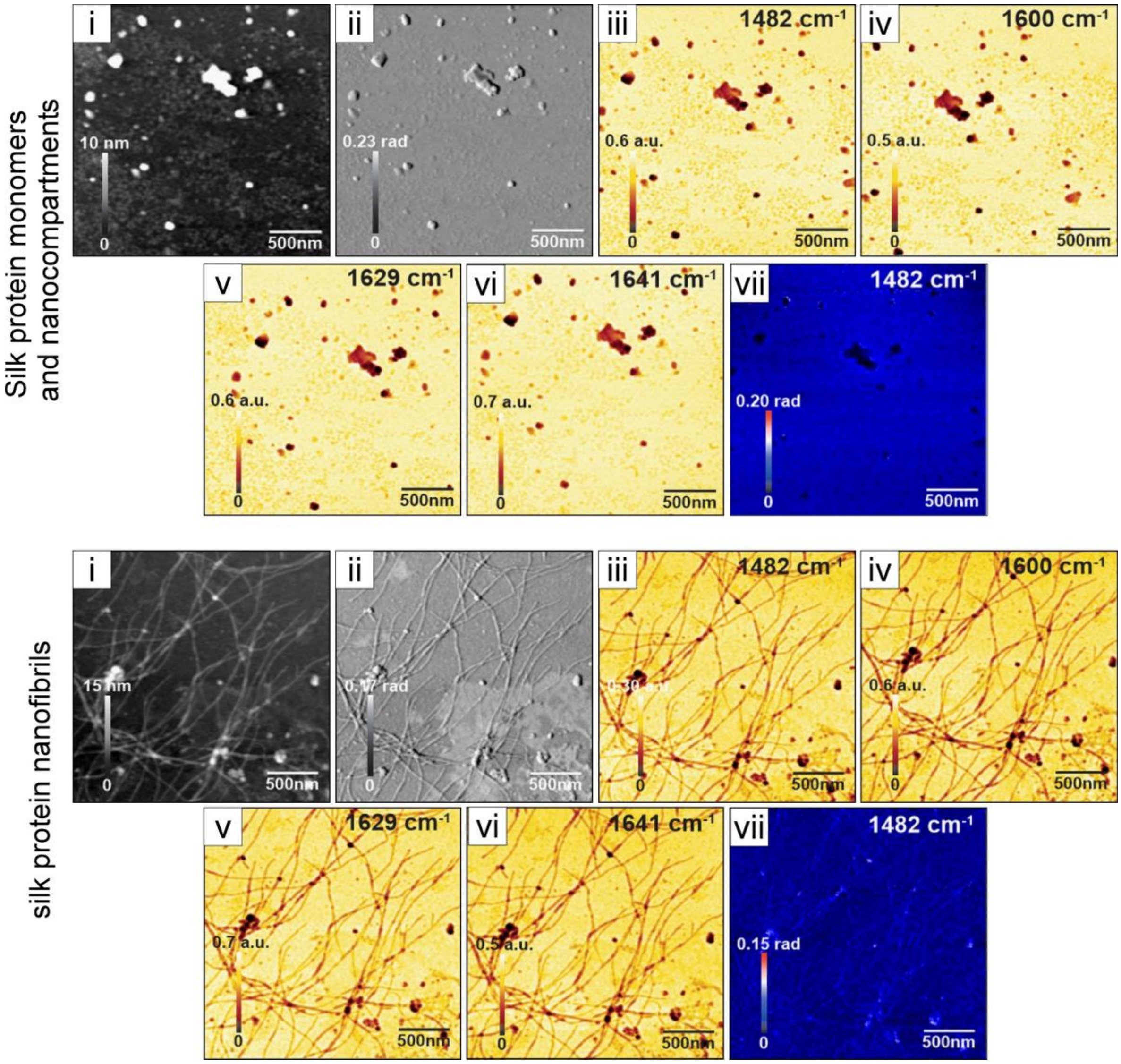

Supplementary Figure 5. Nano-FTIR analysis of silk protein monomers and nanocompartments. Top panel) depicts protein monomers and nanocompartments, bottom panel shows nanofibrils. *(i)* AFM topography images; *(ii)* IR-phase images of silk assemblies; *(iii)* amplitude (reflectance) signal collected at 1482 cm^{-1}; *(iv)* amplitude signal collected at 1600 cm^{-1}; *(v)* amplitude signal collected at 1629 cm^{-1}, used for the evaluation of β-sheet content; *(vi)* Map of the reflection signal collected at 1482 cm^{-1}; *(vii)* FTIR absorption maps collected at 1482 cm^{-1}, which corresponds to C-H bending vibrations, at which the amide region of the protein is invisible and thus used as a negative control. Scale bars: 500 nm.

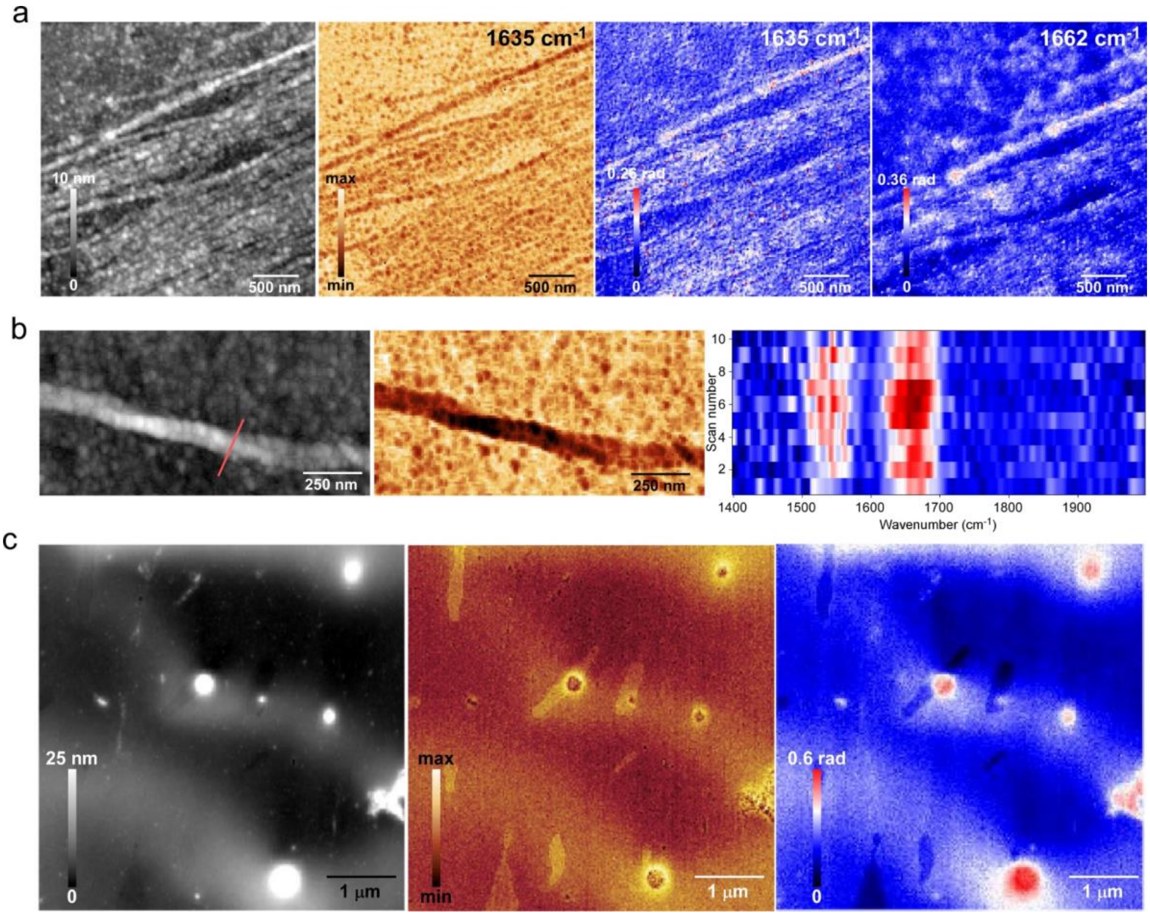

Supplementary Figure 6. Nano-IR analysis of silk protein assemblies. *(a)* From left to right: AFM topography image of silk protein nanofibrils; IR amplitude signal of in protein nanofibrils collected at 1635 cm^{-1}; IR phase map (related to absorbance) of protein nanofibrils at 1635 cm^{-1}; IR phase map of protein nanofibrils at 1662 cm^{-1}. Scale bars: 500 nm. *(b)* From left to right: AFM topography image of a single silk protein nanofibril, IR amplitude signal of a single silk protein at 1635 cm^{-1}; line profile (from left image) of 2D IR spectra collected from a single silk protein nanofibril. Scale bars for AFM images: 250 nm. *(c)* From left to right: topography AFM image of silk protein nanocompartments; IR amplitude signal of protein silk nanocompartments at 1662 cm^{-1}; IR phase map of protein nanocompartments at 1662 cm^{-1}. Scale bars: 1 µm.

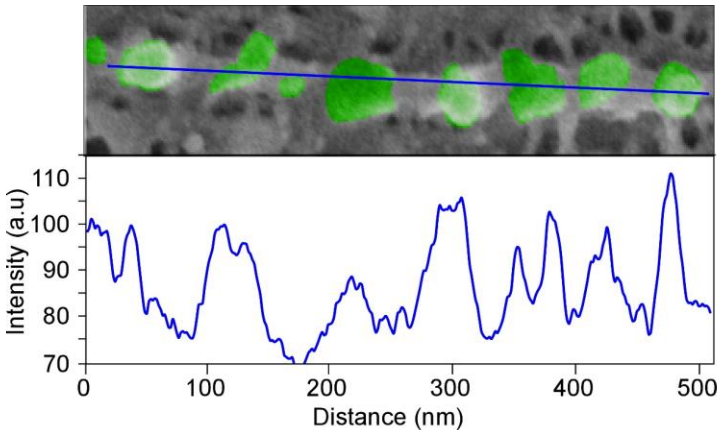

Supplementary Figure 7. Silk nanocopartments comprising silk nanodibril. Freeze-fractured cryo-electron microscopy image (top) of a single silk nanofibril from the anterior part of the silkworm silk gland. The image reveals an event of linear ordering of the fibroin nanocompartment assemblies. Bottom histogram analysis of the size and spacing between the fibroin nanocompartments.

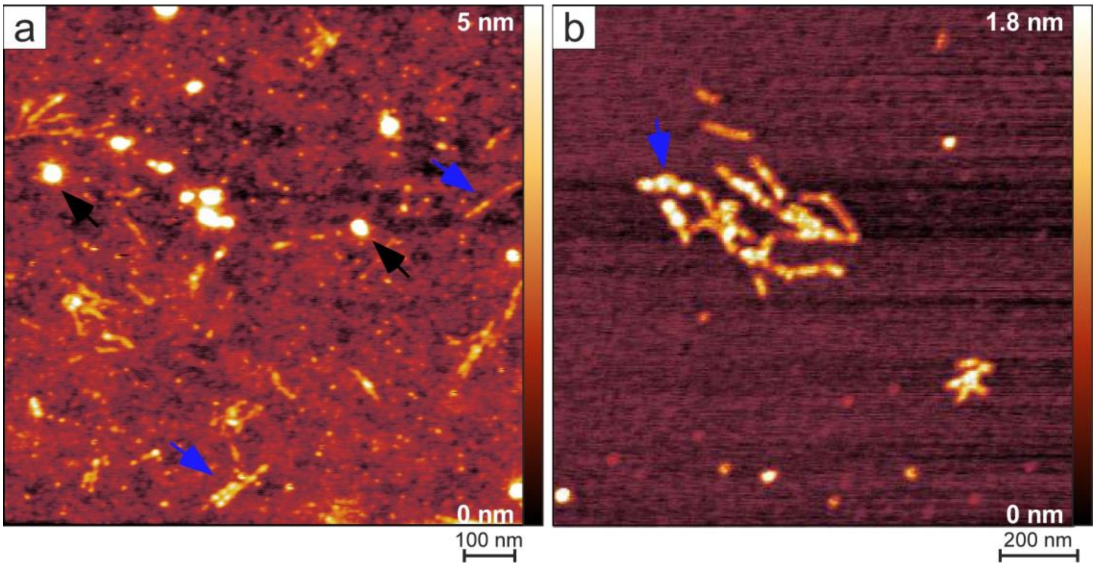

Supplementary Figure 8. Morphology of protein monomers, nanocompartments and nanofibrils. *(a)* AFM topography of nanocompartments (marked with black arrows), nanofibrils (marked with blue arrows) and silk monomers. *(b)* AFM image of nanofibrils composed of nanocompartments of ~50 nm (diameter).

Supplementary Notes

Supplementary Note 1

Nano-FTIR spectroscopy for secondary structure analysis of proteins

As of yet, there is no established procedure to estimate the secondary structure of proteins from nanoscale scattering-type scanning near-field optical microscopy (s-SNOM) spectra in the infrared spectral range. These spectra do not necessarily have the same shape and spectral position as standard FTIR spectra for the following reasons: (i) the incident tip-enhanced electric field is polarized perpendicular to the sample surface[1,2] and, therefore, excites predominantly vibrational transitions with transition dipole moments that point at the same direction. (ii) The band positions of the phase and nano-FTIR absorption spectra obtained at different harmonics of the cantilever oscillation frequency are usually different. This makes it difficult to use standard spectral ranges for the secondary structures to assign band components in the s-SNOM spectra. Effect (i) can be avoided by the random orientation of the proteins on the substrate surface, while effect (ii) can be estimated and approximately corrected for, as outlined in the following.

The band positions of the phase and nano-FTIR absorption spectra obtained at different harmonics of the cantilever oscillation frequency are listed in **Supplementary Table 1**. Those of the phase spectra are always higher than those of the nano-FTIR absorption spectra, a difference that is more pronounced at higher demodulation frequencies. The same trend was observed in spectra of the protein bacteriorhodopsin, the major constituent of the purple membrane of haloarchaea; this observation can be explained by the different probing depths at different harmonics (Paul et al., manuscript in preparation) and by the effect of the sample thickness on the spectra[3].

These band positions are higher than those in our bulk FTIR spectra (**Figure 2**), which will be explained in the following. We will focus on spectra obtained at the 3rd harmonic because these can be compared to the study of sample thickness effects by Mastel et al. [3]. They showed that the band position in bulk FTIR measurements corresponds to the 3rd harmonic nano-FTIR absorption band position of thick films and that the difference between the band positions in the phase and the nano-FTIR absorption spectrum increases with sample thickness. For our samples, this difference ranged from 5 to 9 cm^{-1} for monomers, from 6 to 11 cm^{-1} for nanocompartments, and from 1 to 8 cm^{-1} for fibrils. Thicker samples—for example, fiber bundles—produce larger differences. The

largest difference was 11 cm^{-1}, observed for the spectrum of the largest nanocompartment. This is in good agreement with the thick film results for poly(methyl methacrylate) of Mastel et al. [3,4], who obtained a difference of 9.5 cm^{-1}. Spectra from most of our locations show a difference smaller than 11 cm^{-1}, indicating that they stem from locations that are between the thin film and thick film limits. Assuming 11 cm^{-1} as the thick film limit for our samples, we can now estimate how much higher our observed nano-FTIR absorption band position is than the estimated band position for a thick film. For this we simply subtract the actual difference (see the difference values in bold in **Supplementary Table 1**) from the thick film difference of 11 cm^{-1}.

To correlate our nano-FTIR absorption spectra in **Figure 3** to the bulk FTIR spectra in **Figure 2**, we need to consider also the difference between the nano-FTIR absorption band position obtained at the 2nd harmonic and that obtained at the 3rd harmonic. We evaluated the 2nd harmonic because of its superior signal-to-noise ratio, but data that correlate the nano-FTIR absorption spectra to bulk FTIR spectra are only available for higher harmonics[3,4]. For our nano-FTIR absorption spectra, the 2nd harmonic spectrum has a ~5 cm^{-1} higher band position than the 3rd harmonic, which needs to be taken into account to estimate the corresponding band position in bulk FTIR spectra.

In summary, we estimate a band position corresponding to bulk FTIR measurements for our nano-FTIR spectra by considering (i) the shift between the 2nd and 3rd harmonic nano-FTIR absorption spectra and (ii) the shift between films of intermediate and large thickness. This gives an estimated band position for the 3rd harmonic of a thick film sample that has been shown to correspond to bulk FTIR measurements[3]. We conclude that our nano-FTIR band positions are ~10 cm^{-1} higher than corresponding bulk FTIR band positions. The resulting estimated band positions corresponding to bulk-FTIR spectra are listed in **Supplementary Table 2**. They are in approximate agreement with the bulk-FTIR results for the monomer samples, but are still ~10 cm^{-1} higher for the other samples. This is likely due to the contribution of monomers also to the spectra of these samples, which is supported by the spectra from locations without nanocompartments or nanofibrils, because they also showed amide I absorption. A further reason is the lower spectral resolution of our nano-FTIR absorption spectra, which is expected to make the asymmetric spectra of nanocompartments and nanofibrils more symmetric and in consequence leads to a higher wavenumber of the band position.

Supplementary Tables

Supplementary Table 1

Band positions in phase and nano-FTIR absorption spectra obtained at different harmonics of the cantilever oscillation frequency. Spectra were smoothed over 13 points with OPUS (Bruker, Germany), vector-normalized between 1700 and 1600 cm^{-1} and averaged. Note that the band positions differ from those of the unsmoothed spectra shown in Figure 3. Smoothing was beneficial for this analysis because it removed noise in the spectra at higher harmonics and gave more consistent results between different samples as seen in a smaller standard deviation of the peak positions obtained from different locations. Values were calculated with one digit precision and rounded to integers. Discrepancies between band positions and their differences are due to rounding. nA: nano-FTIR absorption, Diff.: nano-FTIR absorption band position minus phase band position. Some of the latter values are highlighted in bold because they are used to estimate the "true" band position of the nano-FTIR absorption spectra.

	Band position and difference in band position / cm^{-1}								
	Monomers			Nanocompartments			Nanofibrils		
	nA	Phase	Diff.	nA	Phase	Diff.	nA	Phase	Diff.
2nd	1661	1665	-4	1661	1665	-5	1661	1662	-2
3rd	1656	1663	**-7**	1655	1664	**-8**	1657	1661	**-4**
4th	1654	1663	-9	1652	1662	-10	1659	1663	-5
Thick film shift, 3rd harmonic	-4			-3			-8		
Shift from 2nd to 3rd harmonic	-5			-6			-4		
Total shift to estimated bulk FTIR band position	-10			-9			-12		

Supplementary Table 2

Band positions in bulk-FTIR spectra (Figure 2) and in nano-FTIR absorption spectra (Figure 3) obtained at the 2nd harmonic of the cantilever oscillation frequency. Bulk-FTIR band positions were also estimated from the nano-FTIR absorption spectra as described. Spectra were averaged over several locations.

	Band position / cm^{-1}		
	Monomers	Nanocompartments	Nanofibrils
nano-FTIR absorption spectrum, 2nd harmonic, not smoothed	1660	1658	1658
Estimated bulk-FTIR band position from nano-FTIR spectrum	1650	1650	1647
Bulk-FTIR band position (Figure 2)	1647	1638	1620

References

1. Keilmann, F. & Hillenbrand, R. Near-field microscopy by elastic light scattering from a tip. *Philos. Trans. A. Math. Phys. Eng. Sci.* **362**, 787–805 (2004).
2. Amenabar, I. *et al.* Structural analysis and mapping of individual protein complexes by infrared nanospectroscopy. *Nat. Commun.* **4**, (2013).
3. Mastel, S., Govyadinov, A. A., De Oliveira, T. V. A. G., Amenabar, I. & Hillenbrand, R. Nanoscale-resolved chemical identification of thin organic films using infrared near-field spectroscopy and standard Fourier transform infrared references. *Appl. Phys. Lett.* **106**, 023113 (2015).
4. Mester, L., Govyadinov, A. A., Chen, S., Goikoetxea, M. & Hillenbrand, R. Subsurface chemical nanoidentification by nano-FTIR spectroscopy. *Nat. Commun. 2020 111* **11**, 1–10 (2020).
