# Supplementary material for: Micro and nano-scale compartments guide the structural transition of silk protein monomers into silk fibers
Source: Nat Commun. 2022 Dec 21;13:7856. doi: 10.1038/s41467-022-35505-w (PMC9772184; doi:10.1038/s41467-022-35505-w)
Supplement: Supplementary file 2 — Description of Additional Supplementary Files [file 41467_2022_35505_MOESM2_ESM.pdf]

Description of Additional Supplementary Files

Supplementary Movie 1: Video depicting the formation of colloidosomal silk fibroin compartments.

https://www.dropbox.com/s/oz8f6vlsu2lf6d2/Movie1.avi?dl=0

Supplementary Movie 2: Zoomed in video depicting the formation of colloidosomal silk fibroin compartments.

https://www.dropbox.com/s/f7o2n6hynj8a4r8/Movie2.avi?dl=0
